# Coronary magnetic resonance angiography combined with stress-perfusion and delayed-enhancement magnetic resonance imaging for the detection of occult coronary artery disease in asymptomatic individuals

**DOI:** 10.1186/1532-429X-16-S1-P231

**Published:** 2014-01-16

**Authors:** Kyoung Doo Song, Sung Mok Kim, Sang-Chol Lee, Wooin Jung, Sung-A Chang, Yoon Ho Choi, Jidong Sung, Yeon Hyeon Choe

**Affiliations:** 1 Radiology, Samsung Medical Center, Seoul, Korea, Republic of; 2 Cardiology, Samsung Medical Center, Seoul, Korea, Republic of; 3 Center for Health Promotion, Samsung Medical Center, Seoul, Korea, Republic of

## Background

To evaluate the feasibility of using coronary magnetic resonance angiography (CMRA) with stress-perfusion and delayed-enhancement MRI as a screening tool for detection of coronary artery disease in asymptomatic patients.

## Methods

Three hundred forty-one self-referred asymptomatic subjects were enrolled in this study. CMRA image quality and factors affecting image quality were evaluated using a 1.5-T scanner with a 32-channel cardiac coil. Coronary artery stenosis, regional wall motion abnormalities, myocardial perfusion abnormalities, and delayed myocardial enhancement were analyzed. The occurrence of new chest pain and cardiac events was assessed with review of medical records and by telephone interviews in 332 subjects (97.3%) during the 29 ± 6 months (range, 18 to 39 months) follow-up period.

## Results

A total of 3296 (82.4%) of 4000 coronary artery segments exhibited diagnostic image quality on combined whole-heart and volume-targeted MRA. Image quality was affected by heart rates and navigator efficiency in whole-heart CMRA, and heart rates and ages in volume-targeted CMRA. Combined MRI detected significant coronary artery stenosis in eleven (3%) of 341 subjects. Three subjects (0.9%) underwent percutaneous coronary intervention after coronary artery disease was detected on cardiac MRI. There were no cardiac events during the follow-up period in subjects with follow-up.

## Conclusions

Coronary MRA combined with stress-perfusion and delayed enhancement MRI may help to rule out significant coronary artery disease in asymptomatic individuals.

## Funding

No.

